# Supplementary material for: Adapting for the COVID-19 pandemic in Ecuador, a characterization of hospital strategies and patients
Source: PLoS One. 2021 May 17;16(5):e0251295. doi: 10.1371/journal.pone.0251295 (PMC8128267; doi:10.1371/journal.pone.0251295)
Supplement: S1 Fig — (DOCX) [file pone.0251295.s001.docx]

**Title:** Adapting for the COVID-19 pandemic in Ecuador, a characterization of hospital strategies and patients

**Authors:** Daniel Garzon-Chavez, Daniel Romero-Alvarez, Marco Bonifaz, Juan Gaviria, Daniel Mero, Narcisa Gunsha, Asiris Perez, María Garcia, Hugo Espejo, Franklin Espinosa, Edison Ligña, Mauricio Espinel, Emmanuelle Quentin, Enrique Teran, Francisco Mora, Jorge Reyes


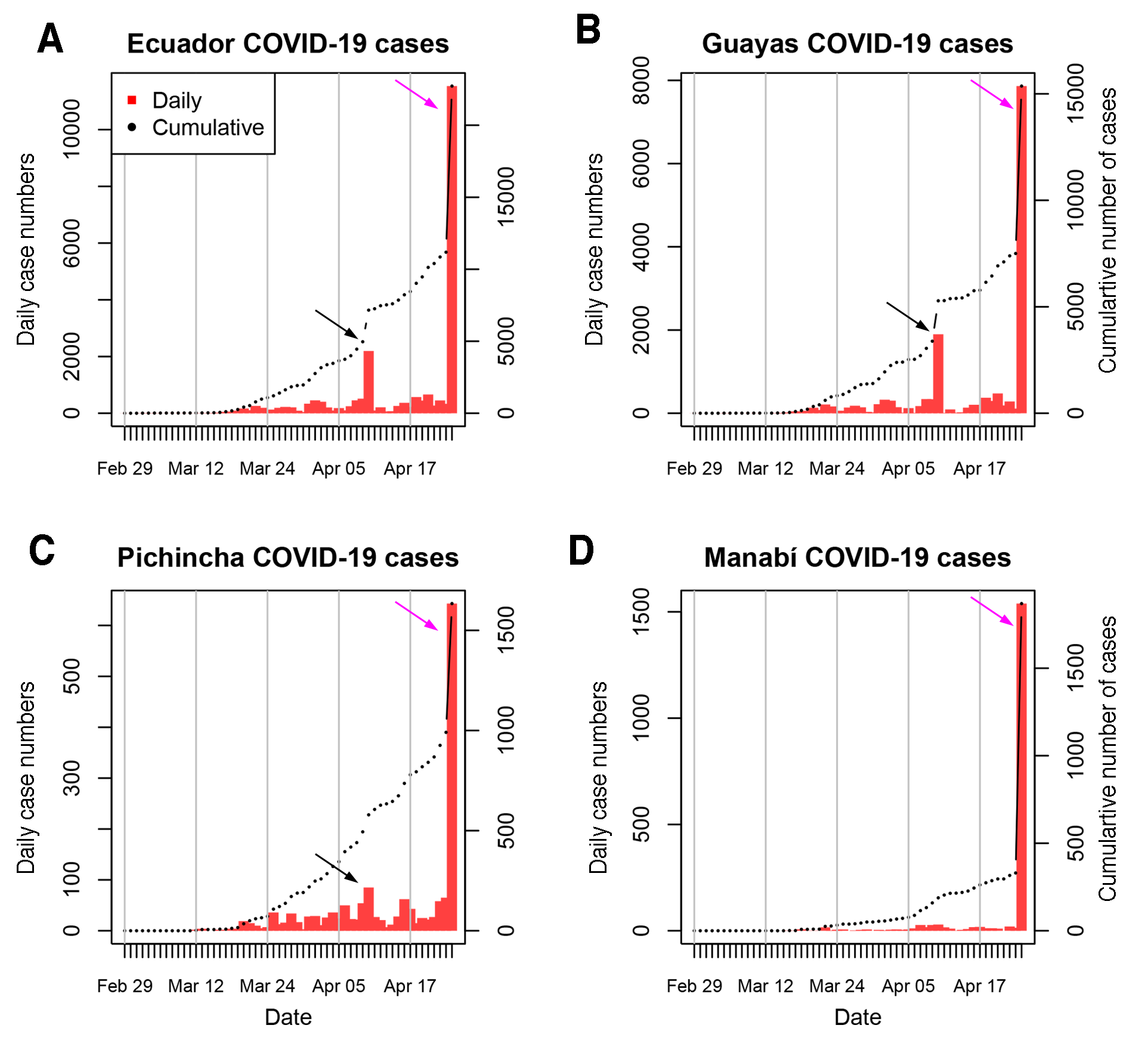


**S1 Fig. Official cumulative and daily case counts of COVID-19 in Ecuador from 29 February to 24 April 2020.** Two remarkable examples of case reporting early in the pandemic can be seen at 10 April, where a release of 2,195 cases (black arrows) showed a sudden spike of COVID-19 in the country followed by reports of 97; 209; and 63 cases the next three days. On 24 April, 11,000 positive cases of COVID-19 were released from one day to the other for a total of 22,719 positive cases, without clear justification on the sudden release (pink arrows). Plots are depicted for the whole country Ecuador (A) and the three most affected provinces: Guayas (B; 15,365 cumulative cases), Pichincha (C; 1,634 cumulative cases), and Manabí (D; 1,869 cumulative cases).
