# Supplementary material for: Adapting for the COVID-19 pandemic in Ecuador, a characterization of hospital strategies and patients
Source: PLoS One. 2021 May 17;16(5):e0251295. doi: 10.1371/journal.pone.0251295 (PMC8128267; doi:10.1371/journal.pone.0251295)
Supplement: S1 Table — (DOCX) [file pone.0251295.s002.docx]

**Title:** Adapting for the COVID-19 pandemic in Ecuador, a characterization of hospital strategies and patients

**Authors:** Daniel Garzon-Chavez, Daniel Romero-Alvarez, Marco Bonifaz, Juan Gaviria, Daniel Mero, Narcisa Gunsha, Asiris Perez, María Garcia, Hugo Espejo, Franklin Espinosa, Edison Ligña, Mauricio Espinel, Emmanuelle Quentin, Enrique Teran, Francisco Mora, Jorge Reyes

**S1 Table. Contingency table evaluating artificial intelligence (AI)-assisted chest computer tomography (CT) system for COVID-19 triage.**

| **AI-assisted chest CT/**  **Confirmatory test** | **>70% CT score** | **<70% CT score** | **Totals** |
| --- | --- | --- | --- |
| Positive RT-PCR | 6 | 22 | 28 |
| Negative RT-PCR | 3 | 6 | 9 |
| Total | 9 | 28 | 37 |
