# Supplementary material for: Adapting for the COVID-19 pandemic in Ecuador, a characterization of hospital strategies and patients
Source: PLoS One. 2021 May 17;16(5):e0251295. doi: 10.1371/journal.pone.0251295 (PMC8128267; doi:10.1371/journal.pone.0251295)
Supplement: Attachment — Submitted filename: PONE-D-20-15485_reviewer comments.docx [file pone.0251295.s007.docx]

**PLOS One Reviewer Comments**

**Manuscript PONE-D-20-15485**: Adapting in middle of COVID-19 pandemic in Ecuador, a Hospital strategies and patients characterization

**Importance**

Clinicians in low- and middle-income countries, in particular those that face health systems constraints, may find the strategies outlined in this paper useful. The mitigation strategies specific to the Ecuadorian context may be implemented by other health facilities in the country.

**Originality**

This is an original study describing management of COVID-19 patients in resource-constrained settings to prevent hospital-acquired infections. The authors described an approach to triaging patients by AI-assisted CT scan score, determining the efficacy of this technology, the clinical characteristics of the first 75 COVID-19 patients, and mapping the potential exposure to healthcare workers for the metropolitan area.

**Clarity**

The manuscript would benefit from a reorganizing and language editing. The description of the creation of the COVID-19 management center should be moved to the introduction section, along with the protocol for triaging patients. The methods section should be devoted strictly to the quantitative methodology (assessment of the sensitivity and specificity of AI-assisted CT software, description of the patients, and geo-distribution of health care workers), as should the results section. This will help focus the paper and provide the necessary contextual information prior to the analysis.

**Comments by manuscript section**

- **Abstract/summary**
  I recommend that the abstract be revised so that it contains all the expected parts of an abstract (brief introduction, purpose/objective, methods, results discussion). The current abstract outlines the purpose of the study but leaves out study results and conclusions. At 178 words, the authors should have room to make use of the full 300 words allotted to include these important findings, such as the sensitivity/specificity of the imaging software to detect true cases, and other salient findings.
- **Research question**
  The research questions were clearly outlined.
- **Overall design of study**

As this is a descriptive study, the chosen methods are appropriate.

- **Introduction**

The general flow of the narrative is good. As I stated previously, I recommend moving the protocol for hospital transformation and patient management into the introduction section. Sub-section headings will be helpful here.

*Specific comments*

- Line 58: Please revise. Is this referring to 97,209 cumulative cases or 97,209 cases reported in the three days following the release of 2,195 cases? The figures do not support the 97,209 number mentioned here.
- Line 60: These figures are not clear. Data are shown on two scales: Bars for daily case counts and dots for cumulative cases. Neither scale goes up to 22,000
- **Methods**
- The authors should briefly describe the study participants, as there are two groups (patients and health care workers). For patients, please clarify the inclusion criteria; several dates were mentioned in the introduction section, but I was not sure what the dates meant. The text (line 157-158) reads, “We considered data from patients either death or discharged admitted in the HGSQ during February 29th to March 28th.” Does the Feb 29-Mar 28 range refer to the admission date or the death/discharge dates?
- The author should briefly outline how they calculated the reliability of the AI-assisted CT scan software.
- Line 149: Unclear what is meant by a “score room,” although further below, in the results, the authors described how patients were separated by score into 3 categories, so I’m assuming that’s what that means. Moving the narrative on patient management and triangulation from the results to the introduction should help clarify things.
- In the sub-section on spatial distribution (beginning line 158), please also include the frequency that information was collected from the health-care workers. Did they complete the online forms daily, weekly, or just once over the study period (lines 163-164)?
- Line 172: Please list the distances for each buffer zone.
- **Results**
- Description of the creation of the COVID-19 management center (lines 194-236) should be moved to the introduction.
- Line 200: Unclear what is meant by “the VM protocol was activated four times.” Does it mean there were 4 patients that needed to be managed in the first 24 hours? Without context/knowing what the average or expected number of activations is (for example—is a higher number good or bad?), this number is somewhat meaningless.
- Line 207 and throughout: Please be consistent in using the term “service” or “department.”

*Sub-section: efficacy of chest CT*

- Line 240 and throughout: Please be consistent in the decimal points chosen when expressing a number. Sometimes 1 decimal point is used and sometimes 2. For percentages, 1 decimal point is enough precision.
- Lines 253-260: There should be a 2 x 2 table to accompany the information in this paragraph.

*Sub-section: Epidemiological characteristics*

- Line 267: Table shows median, not mean, but text says “mean.” Please confirm which one is the correct one.
- Line 270: Figure 1 is referred to in the sentence on the % of people who travelled to Guayaquil, but that figure doesn’t convey that information, Table 1 does. In addition, Supplementary file 1 appears to contain raw data, which is not necessary to refer to in the narrative.
- Line 280 and throughout: The authors use the term “altered” but I think what they meant was “elevated.”

*Sub-section: Health personal contact trace and georeferencing*

- Line 320: Should be “health personnel.”
- Line 321: Median may be more appropriate here than mean.
- Line 324: Not clear if these were all health workers or just the ones who took care of COVID-19 patients.
- **Tables and figures**
- Overall comment: Ensure that the captions of all tables and figures are descriptive enough such that readers can understand the content of the tables and figures without having to read the text. This usually means describing what the table shows (“distribution of…” “sensitivity and specificity of…” etc.), any other variables that are being crosstabbed, the sample/sub-sample and sample size, and dates (if applicable).
- Please use boldface for table superheaders, headers, and sub-headers (within table) so readers can tell easily where different sections of a table starts/ends.
- Removing vertical lines and minimizing the amount of horizontal lines in the table would improve its readability.

Example:

| **Patient characteristics** | **Laboratory test result** | |
| --- | --- | --- |
|  | **Positive**  **n (%)** | **Negative**  **n (%)** |
| XXX |  |  |
| YYY |  |  |
| **Total** |  |  |

*Table 1*

- The caption needs to be revised as the content of this table is unclear. The narrative states there were 75 patients with laboratory-confirmed SARS-CoV-2 infection, the column headers say “positive” and “negative,” so are these referring to the positive or negative determination based on the chest CT scan? If so, this needs to be stated explicitly, as well as the number of people (75).
- The first column needs a header. If these are patient characteristics, that needs to be stated.
- There needs to be a superheader for the column stating exactly what “positive” and “negative” refer to (lab test or CT scan scoring result?).
- Please check the percentages in this table again. For the row on RT-PCR confirmation, that’s clear that row percentages are shown. However for male and female (for example), it is not. 63.9 + 57.1 > 100%, while 57.1 + 42.3 does not add up to 100%.

*Table 2*

- Please revise the caption to be descriptive of the information contained in the table, including the sample size (e.g. “Blood chemical values comparing XXX to XXX among YYYY patients (n=ZZZ), dates”).
- It would be helpful to use thousand separators for the numbers (e.g. 2,000 instead of 2000).

*Table 3*

- Same comment as above to revise the caption to be descriptive of the information contained in the table.
- It would be helpful to use thousand separators for the numbers (e.g. 2,000 instead of 2000).

*Table 4*

- Same comment as above to revise the caption to be descriptive of the information contained in the table.
- See my comment on formatting the tables using boldface and minimize use of lines in the table to make it easier to read. In particular, the sub-sections on ICU and non-ICU patients should be made more prominent.
- The column headers on “remain or transfer to ICU” and “hospital discharged” should have (n)s listed. Please also clarify what “remain” means. Are these patients that remained in their score room? Perhaps the people who remained in their score room should be presented separately from people who were transferred to the ICU (e.g. split the data in this column into two separate columns).
- It may be helpful to list treatment regimens that contain the same medications close to one another, but in the order of increasing medicine. For example, if the treatments can contain medicines A, B, C, and D, list A + B first, then list A + B + C or A + B + D. That way, the common medications are obvious, so other clinicians easily see what other medications are in other regimens.
- It may also be helpful to list these in order of descending frequency if the goal is to show which treatment regimen was most common for ICU and non-ICU patients. Note that this recommendation may conflict with the recommendation immediately above, so the authors should decide on which presentation would be most helpful to other clinicians.

*Figure 1*

- I recommend giving a letter to each of the 4 panels and referring to the panels specifically, for example “Figure 1A,” so that it is clear which one is being referenced.

*Figure 2*

- Ensure correct English spelling of room names

*Figure 3*

- The colors on this map are hard to read, especially for people with color blindness. Recommend using different shapes instead of different colors.
- The hospital marker needs to be more prominent.
- I strongly recommend including the distance for each buffer zone radius somewhere in the figure
- **Discussion and conclusions**
- Overall, the discussion was well-organized, and synthesized information from the results with the current literature. The authors should expand upon their discussion of geographic risk of populations exposed to health care workers caring for COVID-19 patients (lines 432-438), with an emphasis on the population at risk in the metro area.
- The authors should include a paragraph describing the limitations of the current study.
- The manuscript should conclude with a discussion of the strengths of the current study, as well as what future studies should consider.
- **References**
  The references appear to be up-to-date. However, the authors need to ensure that the formatting follows Journal guidelines.
- References for internet sources of information, including news websites, or any official websites (ministry of health, WHO, PAHO, etc.) include the URL and the date that the resource was accessed.
- **Other comments**
- Ensure consistency of spelling throughout (COVID-19 vs COVID19).
- Recommend professional English language editing for grammar and spelling.
- For dates, no comma after month (e.g. “December 2019”, not “December, 2019”).
