# Supplementary material for: Adapting for the COVID-19 pandemic in Ecuador, a characterization of hospital strategies and patients
Source: PLoS One. 2021 May 17;16(5):e0251295. doi: 10.1371/journal.pone.0251295 (PMC8128267; doi:10.1371/journal.pone.0251295)
Supplement: Attachment — Submitted filename: PONE-D-20-15485_revisions_reviewer 1 comments.docx [file pone.0251295.s009.docx]

**PLOS One Reviewer Comments on the Revisions**

**Manuscript PONE-D-20-15485**: Adapting in middle of COVID-19 pandemic in Ecuador, a Hospital strategies and patients characterization

**Overall comments**

I am very pleased with the authors’ responsiveness to my earlier comments. I appreciate the authors’ taking the time to address all of the comments. I believe the flow of the narrative is much clearer after the reorganization. I do not have major concerns, but I have specific grammar comments, as the PLOS One review system asked reviewers to point out those specific errors. This was not something I had looked at closely in the first review. Please be sure to have the English language reviewer do a final pass before you submit your second revisions.

**Comments by manuscript section**

**Abstract/summary**
No further overarching comments

*Specific comments*

- Line 25: Change “overrun” to “overran”

**Introduction**

No further overarching comments

*Specific comments*

- Line 48: Consider changing “rallied” to “amassed”
- Line 49: Should be “case study” instead of “study case”
- Line 53-54: Consider changing this clause: “Guayaquil allowed mass gatherings and delayed strict isolation around two weeks in relation to Quito” to “Guayaquil banned mass gatherings and implemented strict isolation two weeks later than Quito”
- Line 56: Consider changing “Ecuador managed diagnosis of SARS-Cov-2 mainly centralizing real-time reverse transcriptase” to “Ecuador centralized real-time reverse transcriptase”
- Line 92: Change “considering the likelihood” to “with the likelihood”
- Section on hospital distribution for COVID-19 patient attendance (114-135): Recommend capitalizing the names of the clinic area and surgery areas in the narrative and also in Figure 1. For example, instead of “clinical area 1” it should be “Clinical Area 1” so readers understand that it is the formal/proper name of those areas. Same goes for “surgery area 1” 🡪 “Surgery Area 1”
- Line 116: Is “Infection control” a department within the hospital? If so, consider changing to “Infection Control Department”
- Lines 133, 135, and throughout manuscript: Change all instances of “an score” to “a score”
- Line 144: Consider changing “suspected/positive” to “suspected/confirmed”
- Line 148: Change “got infected” to “was infected”

**Methods**

No further overarching comments

*Specific comments*

- Line 183-184: Please clarify: Are these people who had laboratory-confirmed SARS-CoV-2 infection or were these simply people who had the RT-PCR done (regardless of results)?
- Line 190-191: Change “in the context of asymptomatic” to “as asymptomatic”
- Line 193: Here, and throughout, capitalize the proper names of hospital departments, e.g. “Department of Occupational Medicine,” “Infection Control Department”
- Line 196: Change “to suggest potential clusters” to “to identify potential clusters”
- Line 202: Change “and calculated the people at risk” to “and calculated the number of people at risk”

**Results**

No further overarching comments

*Specific comments*

- Line 222: Change “93 positive to SARS-CoV-2” to “93 with laboratory-confirmed SARS-CoV-2 infection”
- Line 225: Change “from both groups” to “overall,” since the overall values are what is being discussed, not the 2 groups separately, as the median was slightly lower for those with a (-) test result
- Line 234: Change “positive patients” to “patients with laboratory-confirmed SARS-CoV-2 infection” or “patients with positive test results” here and throughout. “Positive” is an attribute of the test result or infection status, not of the patients
- Line 236: See comment above. Instead of “positive cases,” use “confirmed cases”
- Line 239: Instead of “negative patients,” use “patients with negative test results”
- Line 299: Change “22-health workers” to “22 health workers”

**Tables and figures**

***Overall comment:***

Perhaps I should have been clearer when I made the recommendation for a more detailed caption. I think the current boldface part of the captions for each table should stay as captions, whereas the non-boldface parts should be table footnotes. What I meant by a more descriptive caption is that captions typically have a descriptor of the sample, the locale (“Quito, Ecuador” or if is the Quito metropolitan area, you may indicate it as “Quito Metropolitan Area, Ecuador”), and some time indicator when the data were collected or sampled (e.g. “February-March 2020”).

For example, for Table 1:

This would be the table caption (note additions highlighted in aqua)

**Table 1. General characteristics, risk factors, and symptoms/signs of the first 75 COVID-19**

**laboratory tested cases attended in the Hospital General del Sur de Quito (HGSQ), Quito, Ecuador -- March 2020**

This would be the table footnote

Cases categorized as positive/negative by laboratory testing (i.e., RT-PCR) from 10 to 28 March 2020.

Percentages from the first row are calculated in relation to the total cases (n = 75). Percentages of the

following rows are calculated with positives, negatives, and totals from the first row, respectively.

For Table 2:

This would be the table caption (note additions highlighted in aqua)

**Table 2. Blood chemical values, laboratory-confirmed COVID-19 cases (n=61) by intensive care unit (ICU) admission status, Quito, Ecuador -- March 2020**

This would be the table footnote

Blood chemical values for 61 COVID-19 positive cases categorized as intensive care unit (ICU; n = 14) and non-ICU (n = 47) from 10 to 28 March 2020. Values represent medians and ranges are depicted in parenthesis. Normal ranges are shown in the headers of each column. CPK = Creatine phosphokinase, LDH = Lactate dehydrogenase, CRP = C-reactive protein, PCT = Procalcitotin, CR = Creatinine; ALT = Alanine transaminase, AST = Aspartate transaminase.

*Table 3*

- Same comment as above to add a locale and brief sample description to the caption, and separate the non-boldface text to the footnote

*Table 4*

- Same comment as above to add a locale and brief sample description to the caption, and separate the non-boldface text to the footnote

*Figure 1 A and B*

- The red arrow on a red box is difficult to see. I recommend changing the color of the boxes to a medium blue (**such as this color**) instead of red and the color of the arrows to black instead of red. Individuals with color blindness may have an especially hard time seeing the red arrows inside the red boxes.

*Figure 2*

No further comments

**Discussion and conclusions**

No further overarching comments

*Specific comments*

- Line 324: Change all instances of “sub-employed”/“sub-employment” to “underemployed”/”underemployment”
- Line 334: Please clarify this line “represents the first attempt to explore the ability of this tool”: the ability of the tool to do what? Also, the first attempt by HGSQ or by whom?
- Line 337: Change “results availability” to “result availability”
- Lines 342-343: Change “poor enough to actually recommend it” to “too poor to recommend”
- Line 343: “adverted” is not the correct word here. Perhaps “cautioned” by the Philippine College of Radiology?
- Line 345: Awkward wording in “bottlenecks of attention.” Perhaps you meant “bottleneck of patients needing attention”
- Lines 351-352: Change “since it is impossible to have the luxury of using the only” to “since we cannot justify using the only”
- Line 353: Change “exposition” to “exposure”
- Line 385: Change “trials)” to “trials”
- Line 394: Should “ICU attendant” be plural (i.e. “ICU attendants”)?
- Line 402: Consider changing “We remark the need” to “Our findings reiterate the need”
- Lines 411-413: Consider changing “Hospitals in low and middle-income countries might follow a similar approach regardless of the scientific evidence in favor or against a particular policy because in emergency contexts, evidence-based information lag with respect to actual response needs.” To “Hospitals in low and middle-income countries might follow a similar approach if there is evidence-based information lag with respect to actual response needs.”
- Line 415: Change “to remark” to “to note”
- Line 416: Change “Health sector in Ecuador is fractionated” to “The health sector in Ecuador is fractured”

**References**

No further comments
