# Supplementary material for: Adapting for the COVID-19 pandemic in Ecuador, a characterization of hospital strategies and patients
Source: PLoS One. 2021 May 17;16(5):e0251295. doi: 10.1371/journal.pone.0251295 (PMC8128267; doi:10.1371/journal.pone.0251295)
Supplement: Attachment — Submitted filename: response to reviewers2.docx [file pone.0251295.s010.docx]

**Response to reviewers 2**

**Title:** Adapting for the COVID-19 pandemic in Ecuador, a characterization of hospital strategies and patients

**Authors:** Daniel Garzon-Chavez, Daniel Romero-Alvarez, Marco Bonifaz, Juan Gaviria, Daniel Mero, Narcisa Gunsha, Asiris Perez, María Garcia, Hugo Espejo, Franklin Espinosa, Edison Ligña, Mauricio Espinel, Emmanuelle Quentin, Enrique Teran, Francisco Mora, Jorge. Reyes

**Reviewer #1**

**Comment 1.** I am very pleased with the authors’ responsiveness to my earlier comments. I appreciate the authors’ taking the time to address all of the comments. I believe the flow of the narrative is much clearer after the reorganization. I do not have major concerns, but I have specific grammar comments, as the PLOS One review system asked reviewers to point out those specific errors. This was not something I had looked at closely in the first review. Please be sure to have the English language reviewer do a final pass before you submit your second revisions.

*Answer: We thank the reviewer for his favorable response to our previous revisions. In this version we have incorporated all the minor changes suggested.*

**Comment 2.** Line 25: Change “overrun” to “overran.

*Answer: Amended (see line 25).*

**Comment 3.** Line 48: Consider changing “rallied” to “amassed”

*Answer: Amended (see line 49).*

**Comment 4.** Line 49: Should be “case study” instead of “study case”

*Answer: Corrected (see line 50).*

**Comment 5.** Line 53-54: Consider changing this clause: “Guayaquil allowed mass gatherings and delayed strict isolation around two weeks in relation to Quito” to “Guayaquil banned mass gatherings and implemented strict isolation two weeks later than Quito”

*Answer: We thank the reviewer for its detailed correction, which we have applied (see lines 54-55).*

**Comment 6.** Line 56: Consider changing “Ecuador managed diagnosis of SARS-Cov-2 mainly centralizing real-time reverse transcriptase” to “Ecuador centralized real-time reverse transcriptase”

*Answer: Correction applied (see lines 57-58).*

**Comment 7.** Line 92: Change “considering the likelihood” to “with the likelihood”

*Answer: Thanks for this suggestion. We decided to not apply this suggestion because in this case, we are talking about three categories of severity and how they are related with COVID-19 positivity, thus, the word ‘considering’ here is clearly relating both sentences (see line 100-104).*

**Comment 8.** Section on hospital distribution for COVID-19 patient attendance (114-135): Recommend capitalizing the names of the clinic area and surgery areas in the narrative and also in Figure 1. For example, instead of “clinical area 1” it should be “Clinical Area 1” so readers understand that it is the formal/proper name of those areas. Same goes for “surgery area 1” 🡪 “Surgery Area 1”

*Answer: We have applied this suggestion in this section, in the figure, and across the main text. Thanks! (see for example lines 132-135 and Fig. 1).*

**Comment 9.** Line 116: Is “Infection control” a department within the hospital? If so, consider changing to “Infection Control Department”.

*Answer: Infection control is a multidisciplinary assembly formed with specialists from different departments. In this version we are calling it Infection Control Unit. We have applied this rewording across the manuscript (see for example line 132 and 169).*

**Comment 10.** Lines 133, 135, and throughout manuscript: Change all instances of “an score” to “a score”.

*Answer: Thank you for noticing this detail. We have corrected it across the manuscript (e.g., see lines 164-165).*

**Comment 11.** Line 144: Consider changing “suspected/positive” to “suspected/confirmed”.

*Answer: Amended (see line 175).*

**Comment 12.** Line 148: Change “got infected” to “was infected”

*Answer: We changed the wording to ‘were infected’ considering the meaning of the entire sentence. Thanks! (see line 179).*

**Comment 13.** Line 183-184: Please clarify: Are these people who had laboratory-confirmed SARS-CoV-2 infection or were these simply people who had the RT-PCR done (regardless of results)?

*Answer: We are referring to the people that had the RT-PCR test done regardless of the results. Notice that from these 75 patients, 61 are laboratory confirmed positive cases and 14 are laboratory confirmed negative, as explained later in the text.*

**Comment 14.** Line 190-191: Change “in the context of asymptomatic” to “as asymptomatic”

*Answer: Changed as suggested (see lines 234-235).*

**Comment 15.** Line 193: Here, and throughout, capitalize the proper names of hospital departments, e.g. “Department of Occupational Medicine,” “Infection Control Department”

*Answer: Amended (e.g., lines 99, 236-247).*

**Comment 16.** Line 196: Change “to suggest potential clusters” to “to identify potential clusters”

*Answer: Fixed (see line 244).*

**Comment 17.** Line 202: Change “and calculated the people at risk” to “and calculated the number of people at risk”

*Answer: Fixed (see line 250).*

**Comment 18.** Line 222: Change “93 positive to SARS-CoV-2” to “93 with laboratory-confirmed SARS-CoV-2 infection”

*Answer: We changed ‘93 positive to SARS-CoV-2’ to ‘93 with potential SARS-CoV-2 infection’ since from this 93, 18 patients passed and the remaining 75 are the ones described in the following lines. Thanks for pointing this out.*

**Comment 19.** Line 225: Change “from both groups” to “overall,” since the overall values are what is being discussed, not the 2 groups separately, as the median was slightly lower for those with a (-) test result.

*Answer: Fixed. Thanks (see line 282).*

**Comment 20.** Line 234: Change “positive patients” to “patients with laboratory-confirmed SARS-CoV-2 infection” or “patients with positive test results” here and throughout. “Positive” is an attribute of the test result or infection status, not of the patients.

*Answer: Thanks for this comment. We have amended this mistake across the manuscript (e.g., 273-275, 318).*

**Comment 21.** Line 236: See comment above. Instead of “positive cases,” use “confirmed cases”.

*Answer: We have amended this wording across the entire manuscript. Thanks! (e.g., 273-275, 318).*

**Comment 22.** Line 239: Instead of “negative patients,” use “patients with negative test results”

*Answer: Thanks. We have fixed the wording in the entire text (e.g., 273-275, 318).*

**Comment 23.** Line 299: Change “22-health workers” to “22 health workers”

*Answer: Fixed (see line 451).*

**Comment 24.** Perhaps I should have been clearer when I made the recommendation for a more detailed caption. I think the current boldface part of the captions for each table should stay as captions, whereas the non-boldface parts should be table footnotes. What I meant by a more descriptive caption is that captions typically have a descriptor of the sample, the locale (“Quito, Ecuador” or if is the Quito metropolitan area, you may indicate it as “Quito Metropolitan Area, Ecuador”), and some time indicator when the data were collected or sampled (e.g. “February-March 2020”).

For example, for Table 1:

This would be the table caption (note additions highlighted in aqua)

**Table 1. General characteristics, risk factors, and symptoms/signs of the first 75 COVID-19**

**laboratory tested cases attended in the Hospital General del Sur de Quito (HGSQ), Quito, Ecuador -- March 2020**

This would be the table footnote

Cases categorized as positive/negative by laboratory testing (i.e., RT-PCR) from 10 to 28 March 2020.

Percentages from the first row are calculated in relation to the total cases (n = 75). Percentages of the

following rows are calculated with positives, negatives, and totals from the first row, respectively.

For Table 2:

This would be the table caption (note additions highlighted in aqua)

**Table 2. Blood chemical values, laboratory-confirmed COVID-19 cases (n=61) by intensive care unit (ICU) admission status, Quito, Ecuador -- March 2020**

This would be the table footnote

Blood chemical values for 61 COVID-19 positive cases categorized as intensive care unit (ICU; n = 14) and non-ICU (n = 47) from 10 to 28 March 2020. Values represent medians and ranges are depicted in parenthesis. Normal ranges are shown in the headers of each column. CPK = Creatine phosphokinase, LDH = Lactate dehydrogenase, CRP = C-reactive protein, PCT = Procalcitotin, CR = Creatinine; ALT = Alanine transaminase, AST = Aspartate transaminase.

*Answer: Thank you very much for your detailed explanation on how to improve the tables. We have applied your suggestions for all the tables (see Tables).*

**Comment 25.** *Table 3**.* Same comment as above to add a locale and brief sample description to the caption, and separate the non-boldface text to the footnote

*Answer: See comment 24.*

**Comment 26.** *Table 4.* Same comment as above to add a locale and brief sample description to the caption, and separate the non-boldface text to the footnote

*Answer: See comment 24.*

**Comment 27.** *Figure 1 A and B.* The red arrow on a red box is difficult to see. I recommend changing the color of the boxes to a medium blue (such as this color) instead of red and the color of the arrows to black instead of red. Individuals with color blindness may have an especially hard time seeing the red arrows inside the red boxes.

*Answer: We agree that the red arrows are going to be difficult to discriminate. We have changed the color of the arrows and lighted colors of the boxes so everything is more noticeable. We decided to avoid changing the color to the suggested blue because the boxes (rooms) represent infectious sources, and therefore dangerous objects, which traditionally are represented with reddish colors. Thanks! (see Fig. 1).*

**Comment 28.** Line 324: Change all instances of “sub-employed”/“sub-employment” to “underemployed”/”underemployment”.

*Answer: Fixed (see line 479).*

**Comment 29.** Line 334: Please clarify this line “represents the first attempt to explore the ability of this tool”: the ability of the tool to do what? Also, the first attempt by HGSQ or by whom?

*Answer: Thank you very much for this comment; we have reworded the sentence (see lines 488-489).*

**Comment 30.** Line 337: Change “results availability” to “result availability”

*Answer: Fixed (see line 492).*

**Comment 31.** Lines 342-343: Change “poor enough to actually recommend it” to “too poor to recommend”

*Answer: Amended (see lines 497-498).*

**Comment 32.** Line 343: “adverted” is not the correct word here. Perhaps “cautioned” by the Philippine College of Radiology?

*Answer: You are totally right. Thank you very much for noticing this miswording. We have changed it as recommended (see line 498).*

**Comment 33.** Line 345: Awkward wording in “bottlenecks of attention.” Perhaps you meant “bottleneck of patients needing attention”

*Answer: We agree with the reviewer, we have changed the sentence (see lines 499-501).*

**Comment 34.** Lines 351-352: Change “since it is impossible to have the luxury of using the only” to “since we cannot justify using the only”

*Answer: Amended (see line 520).*

**Comment 35.** Line 353: Change “exposition” to “exposure”

*Answer: Fixed (see line 522).*

**Comment 36.** Line 385: Change “trials)” to “trials”

*Answer: Done (see line 557).*

**Comment 37.** Line 394: Should “ICU attendant” be plural (i.e. “ICU attendants”)?

*Answer: Thanks for this comment, we were refereeing to the main director of ICU, we have changed it accordingly (see line 571).*

**Comment 38.** Line 402: Consider changing “We remark the need” to “Our findings reiterate the need”

*Answer: Amended (see lines 578-579).*

**Comment 39.** Lines 411-413: Consider changing “Hospitals in low and middle-income countries might follow a similar approach regardless of the scientific evidence in favor or against a particular policy because in emergency contexts, evidence-based information lag with respect to actual response needs.” To “Hospitals in low and middle-income countries might follow a similar approach if there is evidence-based information lag with respect to actual response needs.”

*Answer: Thanks for this comment. We have made the change as suggested (see lines 589).*

**Comment 40.** Line 415: Change “to remark” to “to note”

*Answer: Fixed (see line 591).*

**Comment 41.** 416: Change “Health sector in Ecuador is fractionated” to “The health sector in Ecuador is fractured”

*Answer: Amended. Thanks! (see line 592).*
