# Supplementary material for: Adapting for the COVID-19 pandemic in Ecuador, a characterization of hospital strategies and patients
Source: PLoS One. 2021 May 17;16(5):e0251295. doi: 10.1371/journal.pone.0251295 (PMC8128267; doi:10.1371/journal.pone.0251295)
Supplement: Attachment — Submitted filename: response to reviewers3.docx [file pone.0251295.s011.docx]

**Response to reviewers 3**

**Title:** Adapting for the COVID-19 pandemic in Ecuador, a characterization of hospital strategies and patients

**Authors:** Daniel Garzon-Chavez, Daniel Romero-Alvarez, Marco Bonifaz, Juan Gaviria, Daniel Mero, Narcisa Gunsha, Asiris Perez, María Garcia, Hugo Espejo, Franklin Espinosa, Edison Ligña, Mauricio Espinel, Emmanuelle Quentin, Enrique Teran, Francisco Mora, Jorge. Reyes

Reviewer #1: Dear authors,
I appreciate the effort you have put in to respond to my suggestions in all rounds of reviews. I have reviewed the most recent draft, and I accept the reasons you provided for not making some of the recommended changes.

I have three more minor grammatical changes to recommend in this round that I missed last time:
Line 393 -- There should not be a comma between "trials" and "assessing"
Line 406 -- Change "referred to" to "reported"
Line 421 -- Change "being" to "be"

The paper should be good to go after this. Again, thank you for being so responsive to the comments. This is an important and timely paper not just for this outbreak for future outbreaks.

Warm regards,
Reviewer 1

*Answer: We thank the reviewer for his/her efforts in improve the paper and for his/her comments. In this version we have incorporated all the minor changes suggested and we did a detailed examination of the paper. Also, in Line 402 we change “Deparment” to “Department”.*

*Thank you so much and kind regards.*
